# Catheter ablation vs rate control in atrial fibrillation with left ventricular systolic dysfunction and fibrosis: the CAMERA-MRI II trial

**DOI:** 10.1093/eurheartj/ehaf604

**Published:** 2025-08-29

**Authors:** Louise Segan, Peter M Kistler, Shane Nanayakkara, Andrew Taylor, David M Kaye, James L Hare, Benedict Costello, Hitesh Patel, David Chieng, Rose Crowley, Jeremy William, Hariharan Sugumar, Kenneth Cho, Aleksandr Voskoboinik, Liang-Han Ling, Ziporah Nderitu, Sonia Azzopardi, Annie Curtin, Nikhil Ahluwalia, Malcolm Finlay, Richard Schilling, Kyaw Zaw Win, Manish Kalla, Justin Mariani, Saurabh Kumar, Manuja Premaratne, Ramanathan Parameswaran, John Amerena, Joseph B Morton, Alex J McLellan, Geoffrey Lee, James Theuerle, Stephen Joseph, Michael Wong, Jonathan M Kalman, Sandeep Prabhu

**Affiliations:** Department of Cardiometabolics, The Baker Heart and Diabetes Research Institute, 99 Commercial Rd, Melbourne, VIC 3004, Australia; Department of Cardiology, The Alfred Hospital, 55 Commercial Road, Melbourne 3004, Australia; The University of Melbourne, Parkville, VIC 3010, Australia; Faculty of Medicine, Nursing and Health Sciences, Monash University, 27 Rainforest Walk, Clayton, VIC 3168, Australia; Department of Cardiology, Cabrini Hospital, 181/183 Wattletree Road, Malvern, VIC 3144, Australia; Department of Cardiometabolics, The Baker Heart and Diabetes Research Institute, 99 Commercial Rd, Melbourne, VIC 3004, Australia; Department of Cardiology, The Alfred Hospital, 55 Commercial Road, Melbourne 3004, Australia; The University of Melbourne, Parkville, VIC 3010, Australia; Faculty of Medicine, Nursing and Health Sciences, Monash University, 27 Rainforest Walk, Clayton, VIC 3168, Australia; Department of Cardiology, Cabrini Hospital, 181/183 Wattletree Road, Malvern, VIC 3144, Australia; Department of Cardiometabolics, The Baker Heart and Diabetes Research Institute, 99 Commercial Rd, Melbourne, VIC 3004, Australia; Department of Cardiology, The Alfred Hospital, 55 Commercial Road, Melbourne 3004, Australia; Faculty of Medicine, Nursing and Health Sciences, Monash University, 27 Rainforest Walk, Clayton, VIC 3168, Australia; Department of Cardiology, Cabrini Hospital, 181/183 Wattletree Road, Malvern, VIC 3144, Australia; Department of Cardiometabolics, The Baker Heart and Diabetes Research Institute, 99 Commercial Rd, Melbourne, VIC 3004, Australia; Department of Cardiology, The Alfred Hospital, 55 Commercial Road, Melbourne 3004, Australia; Faculty of Medicine, Nursing and Health Sciences, Monash University, 27 Rainforest Walk, Clayton, VIC 3168, Australia; Department of Cardiology, The Royal Melbourne Hospital, 300 Grattan St, Parkville, VIC 3050, Australia; Department of Cardiometabolics, The Baker Heart and Diabetes Research Institute, 99 Commercial Rd, Melbourne, VIC 3004, Australia; Department of Cardiology, The Alfred Hospital, 55 Commercial Road, Melbourne 3004, Australia; Faculty of Medicine, Nursing and Health Sciences, Monash University, 27 Rainforest Walk, Clayton, VIC 3168, Australia; Department of Cardiometabolics, The Baker Heart and Diabetes Research Institute, 99 Commercial Rd, Melbourne, VIC 3004, Australia; Department of Cardiology, The Alfred Hospital, 55 Commercial Road, Melbourne 3004, Australia; Department of Cardiometabolics, The Baker Heart and Diabetes Research Institute, 99 Commercial Rd, Melbourne, VIC 3004, Australia; Department of Cardiology, Western Health, 176 Furlong Road, St Albans, VIC 3021, Australia; Department of Cardiometabolics, The Baker Heart and Diabetes Research Institute, 99 Commercial Rd, Melbourne, VIC 3004, Australia; Department of Cardiology, The Alfred Hospital, 55 Commercial Road, Melbourne 3004, Australia; Faculty of Medicine, Nursing and Health Sciences, Monash University, 27 Rainforest Walk, Clayton, VIC 3168, Australia; Department of Cardiometabolics, The Baker Heart and Diabetes Research Institute, 99 Commercial Rd, Melbourne, VIC 3004, Australia; Department of Cardiology, The Alfred Hospital, 55 Commercial Road, Melbourne 3004, Australia; The University of Melbourne, Parkville, VIC 3010, Australia; Faculty of Medicine, Nursing and Health Sciences, Monash University, 27 Rainforest Walk, Clayton, VIC 3168, Australia; Department of Cardiology, Cabrini Hospital, 181/183 Wattletree Road, Malvern, VIC 3144, Australia; Department of Cardiometabolics, The Baker Heart and Diabetes Research Institute, 99 Commercial Rd, Melbourne, VIC 3004, Australia; Department of Cardiology, The Alfred Hospital, 55 Commercial Road, Melbourne 3004, Australia; The University of Melbourne, Parkville, VIC 3010, Australia; Faculty of Medicine, Nursing and Health Sciences, Monash University, 27 Rainforest Walk, Clayton, VIC 3168, Australia; Department of Cardiology, Cabrini Hospital, 181/183 Wattletree Road, Malvern, VIC 3144, Australia; Department of Cardiometabolics, The Baker Heart and Diabetes Research Institute, 99 Commercial Rd, Melbourne, VIC 3004, Australia; Department of Cardiology, The Alfred Hospital, 55 Commercial Road, Melbourne 3004, Australia; The University of Melbourne, Parkville, VIC 3010, Australia; Faculty of Medicine, Nursing and Health Sciences, Monash University, 27 Rainforest Walk, Clayton, VIC 3168, Australia; Department of Cardiology, Cabrini Hospital, 181/183 Wattletree Road, Malvern, VIC 3144, Australia; Department of Cardiometabolics, The Baker Heart and Diabetes Research Institute, 99 Commercial Rd, Melbourne, VIC 3004, Australia; Department of Cardiology, The Alfred Hospital, 55 Commercial Road, Melbourne 3004, Australia; The University of Melbourne, Parkville, VIC 3010, Australia; Faculty of Medicine, Nursing and Health Sciences, Monash University, 27 Rainforest Walk, Clayton, VIC 3168, Australia; Department of Cardiometabolics, The Baker Heart and Diabetes Research Institute, 99 Commercial Rd, Melbourne, VIC 3004, Australia; Department of Cardiology, The Alfred Hospital, 55 Commercial Road, Melbourne 3004, Australia; The University of Melbourne, Parkville, VIC 3010, Australia; Department of Cardiometabolics, The Baker Heart and Diabetes Research Institute, 99 Commercial Rd, Melbourne, VIC 3004, Australia; Department of Cardiology, The Alfred Hospital, 55 Commercial Road, Melbourne 3004, Australia; The University of Melbourne, Parkville, VIC 3010, Australia; Faculty of Medicine, Nursing and Health Sciences, Monash University, 27 Rainforest Walk, Clayton, VIC 3168, Australia; Department of Cardiology, Cabrini Hospital, 181/183 Wattletree Road, Malvern, VIC 3144, Australia; Department of Cardiometabolics, The Baker Heart and Diabetes Research Institute, 99 Commercial Rd, Melbourne, VIC 3004, Australia; Department of Cardiology, The Alfred Hospital, 55 Commercial Road, Melbourne 3004, Australia; The University of Melbourne, Parkville, VIC 3010, Australia; Faculty of Medicine, Nursing and Health Sciences, Monash University, 27 Rainforest Walk, Clayton, VIC 3168, Australia; Department of Cardiology, St Vincent’s Private Hospital Fitzroy, 50 Victoria Parade, Fitzroy, VIC 3065, Australia; Department of Cardiometabolics, The Baker Heart and Diabetes Research Institute, 99 Commercial Rd, Melbourne, VIC 3004, Australia; Department of Cardiology, The Alfred Hospital, 55 Commercial Road, Melbourne 3004, Australia; Department of Cardiometabolics, The Baker Heart and Diabetes Research Institute, 99 Commercial Rd, Melbourne, VIC 3004, Australia; Department of Cardiology, The Alfred Hospital, 55 Commercial Road, Melbourne 3004, Australia; Department of Cardiometabolics, The Baker Heart and Diabetes Research Institute, 99 Commercial Rd, Melbourne, VIC 3004, Australia; Department of Cardiology, The Alfred Hospital, 55 Commercial Road, Melbourne 3004, Australia; Department of Cardiology, St Bartholomew’s Hospital, W Smithfield, London EC1A 7BE, UK; Department of Cardiology, St Bartholomew’s Hospital, W Smithfield, London EC1A 7BE, UK; Department of Cardiology, St Bartholomew’s Hospital, W Smithfield, London EC1A 7BE, UK; Department of Cardiology, Queen Elizabeth Hospital Birmingham, Mindelsohn Way, Birmingham B15 2GW, UK; Department of Cardiology, Queen Elizabeth Hospital Birmingham, Mindelsohn Way, Birmingham B15 2GW, UK; Department of Cardiometabolics, The Baker Heart and Diabetes Research Institute, 99 Commercial Rd, Melbourne, VIC 3004, Australia; Department of Cardiology, The Alfred Hospital, 55 Commercial Road, Melbourne 3004, Australia; Department of Cardiology, St Vincent’s Private Hospital Fitzroy, 50 Victoria Parade, Fitzroy, VIC 3065, Australia; Department of Cardiology, Westmead Hospital, Sydney, Australia; Department of Cardiology, Frankston Hospital, Melbourne, Australia; The University of Melbourne, Parkville, VIC 3010, Australia; Department of Cardiology, University Hospital Geelong, Melbourne, Australia; Department of Cardiology, University Hospital Geelong, Melbourne, Australia; The University of Melbourne, Parkville, VIC 3010, Australia; Department of Cardiology, The Royal Melbourne Hospital, 300 Grattan St, Parkville, VIC 3050, Australia; The University of Melbourne, Parkville, VIC 3010, Australia; Department of Cardiology, The Royal Melbourne Hospital, 300 Grattan St, Parkville, VIC 3050, Australia; Department of Cardiology, St Vincent’s Private Hospital Fitzroy, 50 Victoria Parade, Fitzroy, VIC 3065, Australia; The University of Melbourne, Parkville, VIC 3010, Australia; Department of Cardiology, The Royal Melbourne Hospital, 300 Grattan St, Parkville, VIC 3050, Australia; Department of Cardiology, Melbourne Private Hospital, Melbourne, Australia; Department of Cardiology, The Alfred Hospital, 55 Commercial Road, Melbourne 3004, Australia; Department of Cardiology, Western Health, 176 Furlong Road, St Albans, VIC 3021, Australia; Department of Cardiology, Austin Hospital, 145 Studley Road, Heidelberg, VIC 3084, Australia; Department of Cardiology, Cabrini Hospital, 181/183 Wattletree Road, Malvern, VIC 3144, Australia; Department of Cardiology, Western Health, 176 Furlong Road, St Albans, VIC 3021, Australia; The University of Melbourne, Parkville, VIC 3010, Australia; Department of Cardiology, The Royal Melbourne Hospital, 300 Grattan St, Parkville, VIC 3050, Australia; Department of Cardiology, Western Health, 176 Furlong Road, St Albans, VIC 3021, Australia; The University of Melbourne, Parkville, VIC 3010, Australia; Department of Cardiology, The Royal Melbourne Hospital, 300 Grattan St, Parkville, VIC 3050, Australia; Department of Cardiology, Melbourne Private Hospital, Melbourne, Australia; Department of Cardiometabolics, The Baker Heart and Diabetes Research Institute, 99 Commercial Rd, Melbourne, VIC 3004, Australia; Department of Cardiology, The Alfred Hospital, 55 Commercial Road, Melbourne 3004, Australia; The University of Melbourne, Parkville, VIC 3010, Australia; Department of Cardiology, Mulgrave Private Hospital, 48 Blanton Drive, Mulgrave, VIC 3170, Australia; Department of Cardiology, Epworth Hospital Richmond, 89 Bridge Road, Richmond, VIC 3121, Australia

**Keywords:** Atrial fibrillation, Left ventricular systolic dysfunction, Catheter ablation, Left ventricular fibrosis, Late gadolinium enhancement, Rhythm control

## Background

Catheter ablation (CA) for atrial fibrillation (AF) and left ventricular systolic dysfunction (LVSD) is associated with left ventricular ejection fraction (LVEF) improvement, fewer heart failure (HF) hospitalizations, and improved survival^1–3^ and is a class I recommendation as first-line rhythm control for AF-mediated cardiomyopathy.^4,5^

Nonetheless, it remains unclear to what extent pre-existing structural heart disease can impede LV recovery following sinus rhythm (SR) restoration. The CAMERA-MRI I study demonstrated a diminished, albeit significant improvement in LVEF in non-ischaemic CM with fibrosis,^2^ yet CASTLE-HTx study demonstrated significant clinical benefits in patients with end-stage HF with presumed high fibrosis burdens.^6^

CAMERA-MRI II was designed to prospectively explore the impact of LV fibrosis on CA outcomes in patients with AF and LVSD compared with medical therapy.

## Methods

This was an international multicentre randomized controlled trial of patients with AF, LVEF ≤ 45% on cardiac MRI (CMR), and LV fibrosis [late gadolinium enhancement (LGE) ≥ 5%] randomized 1:1 to CA or medical rate control (MRC). Participants were followed for 12 months. The primary outcome was change in CMR LVEF from baseline to 12 months (trial registration: ACTRN12620000502932).

Patients underwent a 5-week medical optimization of rate control and HF pharmacotherapy prior to screening CMR. Follow-up CMR was performed at 12 months—timed from randomisation in the medical therapy arm and from the date of ablation in the CA arm. The CMR protocol has been previously described.^2^ The LV LGE was quantified as a percentage of the total myocardium using a published methodology.^2^ Late gadolinium enhancement positive status was defined as LGE burden ≥ 5% as this threshold has been associated with LVEF non-recovery in patients with AF.^7^ Left ventricular ejection fraction and LGE quantification were centralised in a core lab with two experienced CMR cardiologists, blinded to allocation.

The CA procedure has been published previously.^8^ Pulmonary vein isolation was mandatory; additional ablation was performed at the operator’s discretion. Radiofrequency ablation was utilized in all cases. Medical rate control was assessed using 24 h Holter monitoring at baseline, 3, 6, and 12 months.

Rhythm monitoring comprised implantable cardiac device interrogation (if present) or twice-daily ECG transmissions via a Kardia™ device. Arrhythmia recurrence was defined as any atrial arrhythmia (AF, atrial flutter, or atrial tachycardia) lasting ≥30 s after a 90-day blanking period post ablation.^8^ Atrial fibrillation burden was defined as the proportion of time spent in AF during the 12-month monitoring period, expressed as a percentage.^8,9^

To detect a minimum absolute LVEF change > 6.8% between the LGE positive CA and MRC groups based on a sub-analysis of LGE positive patients from the CAMERA-MRI study,^2^ an estimated sample size of 80 patients (40 per group) was required for statistical power of 80% with the probability of a type one error of 0.05 and accounting for a 10% drop out rate. All analyses were performed using R (version 4.2.0, R Core Team).

## Results

### Baseline characteristics

**Figure 1:**
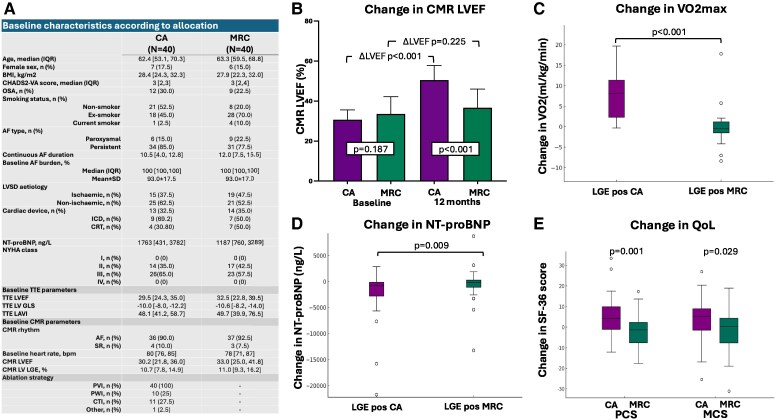
Baseline characteristics and outcomes following catheter ablation vs medical rate control in patients with atrial fibrillation and left ventricular systolic dysfunction with left ventricular fibrosis. (*A*) Baseline characteristics according to allocation. At 12 months, catheter ablation was associated with a significant improvement in left ventricular ejection fraction (*B*), accompanied by improvements in functional capacity (*C*), N-terminal pro-B-type natriuretic peptide (NT-proBNP) (*D*), and quality of life (*E*). AF, atrial fibrillation; CA, catheter ablation; MRC, medical rate control; IQR, interquartile range; BMI, body mass index; OSA, obstructive sleep apnoea; LVSD, left ventricular systolic dysfunction; ICD, implantable cardioverter-defibrillator; CRT, cardiac resynchronization therapy device; NYHA, New York Heart Association; TTE, transthoracic echocardiogram; LVEF, left ventricular ejection fraction; LV GLS, left ventricular global longitudinal strain; LAVI, left atrial volume index; CMR, cardiac magnetic resonance imaging; SR, sinus rhythm; LGE, late gadolinium enhancement; VO2max, maximal volume of oxygen; QoL, quality of life; SF-36, Short Form Survey; PCS, Physical Component Summary; MCS, Mental Component Summary

Between September 2020 and June 2024, 224 patients were assessed and 80 LGE positive individuals [median LGE burden 11% (7, 15), ischaemic aetiology 43%, persistent AF 81%] were randomized to CA or MRC. The median follow-up was 14.3 months (IQR 13.7, 15.2). Baseline characteristics were comparable including NYHA class (NYHA III in 65% in CA and 57.5% in MRC, *P* = .491), AF history (time from AF diagnosis to study enrolment) 18 [9, 36] vs 18 [9, 38] months in MRC, *P* = .852), and baseline AF rate control [CA: 80 b.p.m. (IQR 76, 85); MRC: 72 b.p.m. (IQR 71, 87); *P* = .443; *Figure 1A*]. Among the MRC group, two individuals underwent CA during follow-up.

### Outcomes according to randomization

At 12 months, LGE positive individuals who underwent CA experienced a significantly greater improvement in LVEF [+20% (11, 28)] compared with MRC [+4% (0, 8), *P* < .001, *Figure 1B*], with corresponding improvements in functional capacity [+8.1 mL/kg/min (2.3, 11.4) vs −0.5 mL/kg/min (−1.5, 1.2) in MRC, *P* < .001], reductions in NT-proBNP [−812 ng/L (−2771, −112) vs −124 (−1075, +286) in MRC, *P* = .009], reverse LA remodelling [ΔLAVI −10 mL/m^2^ (−16, 0) vs +6 mL/m^2^ (−6, 22) in MRC, *P* < .001], and improvements in HF symptoms [ΔMLHFQ: −14 (−25, −3) vs −1 (−8, 14), *P* < .001] and quality of life [ΔSF-36 PCS CA: +4 (−1, 10) vs −1 (−8,+2) in MRC, *P* = .001; ΔSF-36 MCS CA: +5 (−1, 9) vs +0 (−8, 4) in MRC; *P* = .029; *Figure 1C–E*]. A *post hoc* ANCOVA adjusting for baseline LVEF confirmed a significant between-group difference in 12-month LVEF (*P* < .001).

There was an inverse correlation between LGE burden and LVEF improvement (*R* = −0.464, *P* = .003) and an attenuation in LVEF improvement with LGE burden ≥ 20% [+5% (3, 7)] compared with lower LGE burden [LGE < 20%: +22% (13, 28), *P* < .001].

## Discussion

In this study, SR restoration achieved via CA resulted in a substantial improvement in LVEF (+20%) at 12 months compared with MRC (4%), accompanied by significant improvements in functional status, HF biomarkers, and quality of life. Although substantial, this improvement attenuated as ventricular fibrosis increased.

While the scar burden in this study was modest, it was notably higher than other published studies (13% vs 7%)^7^ and reflective of a contemporary heterogeneous HF population presenting for rhythm control. The pronounced LVEF improvement may reflect differences in patient selection, including shorter AF duration, higher LVEF inclusion criteria, and modest LV fibrosis burden, in whom there may be less advanced cardiac remodelling and more reversibility in myocardial dysfunction.

Differences in rhythm between serial CMR imaging may have magnified the difference in LVEF between randomized groups. Although CMR assessors were blinded to treatment allocation, rhythm at the time of CMR imaging could not be concealed. Secondary outcomes analyses should be considered hypothesis-generating.

Despite contemporary guidelines recommending CA as a class I indication for rhythm control in AFCM (class IIa for AF in broader LVSD populations),^5^ this treatment modality remains significantly underutilized.^10^ This study highlights the potential for significant reverse remodelling following SR restoration, even in the setting of pre-existing SHD, suggesting that despite pre-existing fibrosis, the degree of reversible myocardial dysfunction attributable to AF is underappreciated.

Furthermore, while a higher baseline LV fibrosis burden may limit the effectiveness of CA, such patients may still derive other clinical benefits such as improved subjective and objective functional capacity. Further studies could explore the threshold of fibrosis beyond which the benefits of CA become negligible, helping to better define the role of CA in patients with LVSD and advanced LV fibrosis. Nonetheless, in light of these findings, the authors propose that, in the absence of contraindications, the presence of ventricular fibrosis in itself should not preclude consideration of rhythm control with CA in patients with LVSD.

## Declarations

### Disclosure of Interest

L.S. has received a combined National Heart Foundation/National Health and Medical Research Council (NHMRC) PhD scholarship. P.M.K. is a recipient of the investigator grant from the NHMRC and has received funding from Abbott Medical for consultancy and speaking engagements and has served on the advisory board with fellowship support from Biosense Webster. J.M.K. has received fellowship support from Medtronic and Biosense Webster. G.L. has received consulting fees from Biosense Webster. S.P. is the recipient of investigator research grants from NHMRC and has received fellowship and training support from the National Heart Foundation, Abbott Medical, and Boston Scientific and has also received speaker fees and advisory fees from Abbott Medical and Biosense Webster.

## Data Availability

Data are available upon reasonable request to the corresponding author.

## Pre-registered Clinical Trial Number

ACTRN12620000502932.
